# Continuous fluid infusion per rectum compared with intravenous fluid infusion in pigs

**DOI:** 10.1111/jvim.16733

**Published:** 2023-05-24

**Authors:** Munashe Chigerwe, Sarah J. Blasczynski, Bailey A. Abi‐Nader, Paige M. Condy, Cileah M. Kretsch, Sarah M. Depenbrock

**Affiliations:** ^1^ Department of Veterinary Medicine and Epidemiology University of California Davis, School of Veterinary Medicine Davis California USA; ^2^ William R. Pritchard Veterinary Medical Teaching Hospital University of California Davis, School of Veterinary Medicine Davis California USA

**Keywords:** albumin, catheter, packed cell volume, porcine, proctoclysis, serum total solids

## Abstract

**Background:**

Peripheral blood vessels in pigs are not easily accessible, making placement of intravenous catheters difficult. Alternative methods to intravenous administration of fluids, such as administering fluids via the rectum (proctoclysis), are warranted in pigs.

**Hypothesis:**

Administration of polyionic crystalloid fluids via proctoclysis results in hemodilution changes similar to intravenous administration. The objectives of this study were to evaluate the tolerance for proctoclysis in pigs and compare analytes before and after intravenous or proctoclysis therapy.

**Animals:**

Six healthy, growing, academic institution‐owned pigs.

**Methods:**

Randomized, cross‐over design clinical trial, with 3 treatments (control, intravenous, and proctoclysis) with a 3‐day washout period. The pigs were anesthetized and jugular catheters were placed. A polyionic fluid (Plasma‐Lyte A 148) was administered at 4.4 mL/kg/h during the intravenous and proctoclysis treatments. Laboratory analytes, including PCV, plasma, and serum total solids, albumin, and electrolytes were measured over 12 h at T_0_, T_3_, T_6_, T_9_, and T_12_. Effects of treatment and time on analytes were determined by analysis of variance.

**Results:**

Proctoclysis was tolerated by pigs. Albumin concentrations decreased during the IV treatment between T_0_ and T_6_ (least square mean of 4.2 vs 3.9 g/dL; 95% CI of mean difference = −0.42, −0.06; *P* = .03). Proctoclysis did not significantly affect any laboratory analytes at any time points (*P* > .05).

**Conclusions and Clinical Importance:**

Proctoclysis did not demonstrate hemodilution similar to intravenous administration of polyionic fluids. Proctoclysis might not be an effective alternative to the intravenous administration of polyionic fluids in healthy euvolemic pigs.

**Abbreviations**
95% CI: 95% confidence interval
CONT: control group treatment
IV: intravenous administration treatment
RECT: per rectum fluid administration treatment

## 1 INTRODUCTION

The placement of intravenous catheters for administering fluids to manage various medical and surgical disease conditions is a standard procedure performed in sick pigs presented for hospital care. Handling and restraining pigs for placement of intravenous catheters is challenging because pigs are excitable, might bite, and are usually unwilling to tolerate such manipulation. Although fractious sick pigs can be sedated or anesthetized for the placement of intravenous catheters, adverse outcomes associated with sedation or anesthesia, such as respiratory impairment including decreased ventilation, aspiration, or death, might occur. While orogastric or nasogastric administration of fluids is clinically valuable and safe for use in horses and ruminants, the method is not tolerated by awake pigs and is unsafe in anesthetized pigs. Therefore, effective and less stressful alternative methods to intravenous administration of fluids, such as administering fluids via the rectum (proctoclysis), are warranted in pigs.

Proctoclysis is technically simple to perform and requires similar equipment as an enema. Except in scenarios where rapid fluid volume resuscitation is required, proctoclysis might be an inexpensive, safe, effective, and less stressful alternative to intravenous fluid administration. Proctoclysis is used effectively in rabbits,^1^ elephants,^2^ horses,^3^ and humans in end‐of‐life palliative care or remote environments.^4^, ^5^, ^6^ Proctoclysis is accepted as a method for fluid administration in other species; however, limited studies are available describing its efficacy in pigs. Therefore, the objectives of this study were to evaluate the tolerance of fluid administration via the rectum in pigs and compare clinical laboratory variables before and after initiating fluid therapy between the intravenous and per rectum routes. We hypothesized that administering polyionic fluids via proctoclysis in euhydrated pigs results in hemodilution changes similar to intravenous administration.

## 2 MATERIALS AND METHODS

### 2.1 Animals and study design

The sample size was estimated to detect a change in PCV of at least 5%, using an anecdotal mean PCV of 30% in healthy pigs to 25%, after initiation of fluid therapy by either route, similar to studies in horses,^3^ with a PCV SD of 1%, a type 1 error (α) of 0.05, and a power (1‐β) of 80%. The minimum sample required was 5 pigs. To account for a 10% dropout rate due to missing values, 6 pigs were enrolled. The sample size was calculated using JMP Pro v16 software (SAS Institute, Cary, North Carolina). The research was approved by the University of California Davis, Institutional Animal Care and Use Protocol (#21935).

A clinical trial with 6 healthy, growing pigs was performed. Five pigs were Yorkshire barrows, and 1 was a Hampshire gilt. The study was a cross‐over design with 3 treatments and a 3‐day washout period between treatments. The 3‐day washout was chosen based on the half‐life of crystalloid fluids in humans undergoing anesthesia and surgery.^7^ The half‐life of crystalloid fluids is approximately the duration of the anesthesia and surgery and ranges from 3 to 8 hours.^7^ The pigs were randomly assigned to 1 of 3 treatments at a time; control (CONT), administration of fluids via the rectum (RECT), and administration of fluids IV. A clinical examination was performed at enrollment. The pigs were housed individually in pens measuring 3 × 3 m. The pigs were allowed to acclimatize to the study environment for 2 days. During the acclimatization period, pigs were fed 2 kg of commercial pig feed twice daily, and water was available ad libitum. Two pigs were enrolled at a time.

### 2.2 Placement of intravenous catheters

Before placing catheters, feed and water were withheld from the pigs for 12 and 8 hours, respectively. Anesthesia was induced with a combination of tiletamine and zolazepam (Telazol, Zoetis, Parsippany, New Jersey) administered intramuscularly. Anesthesia was maintained with isoflurane (Fluriso, MWI, Boise, Idaho) dosed to effect (1%‐5%) and oxygen (4 L/min) using a mask. The pigs were monitored for depth of anesthesia by using a capnometer and monitoring of heart rate, respiratory rate, mucus membrane color, and capillary refill time by trained personnel. The pigs were positioned in dorsal recumbency, and the forelimbs extended caudally and secured on the surgical table with soft ropes. An area measuring 15 × 15 cm on the ventral and lateral left and right neck was clipped to expose the area over the jugular groove and aseptically prepared for the intravenous catheter placement. An over‐the‐wire 2‐ or 3‐port, 4 French × 13 cm, long‐term intravenous catheter (Long‐term intravenous catheter, Mila International, Florence, Kentucky) was placed in the right and left jugular vein as previously described.^8^ Briefly, a fenestrated sterile drape was placed over the surgical site, and a 7‐8 cm craniolateral to caudomedial incision was made over the jugular groove. The subcutaneous and muscular tissues were bluntly dissected to expose the jugular vein. Weitlaner self‐retaining retractors were used to maintain exposure of the jugular vein. The tissue around the jugular vein was gently dissected to free the vein. Two Covalt's spay hooks were used to elevate the jugular vein to the skin surface, followed by the placement of the catheter. The subcutaneous tissue was closed using size 2‐0 absorbable polydioxanone suture material (PDS II, Ethicon, Inc, Raritan, New Jersey) in a simple continuous pattern. The skin was closed using size 0 nonabsorbable polypropylene suture material (Prolene, Ethicon, Inc, Raritan, New Jersey) in a simple interrupted pattern. The catheter placement procedure was repeated on the opposite jugular vein. The catheters were secured to the skin using size 0, nonabsorbable polypropylene suture material, and an adhesive bandage wrap (Elastikon, Johnson and Johnson, New Brunswick, New Jersey). Once the catheters were placed, CBC and serum biochemical analysis were performed to confirm health status. One intravenous catheter was assigned for blood collection, whereas the second was assigned for fluid administration. The pigs were then recovered from anesthesia. Intravenous catheter patency was achieved by flushing with 0.45‐0.7 mL heparinized saline (1:10 dilution) every 24 hours. The intravenous catheter site was monitored for swelling, redness, pain on palpation, and discharge (blood, serosanguineous fluid, or pus), twice daily. Treatment procedures were performed 48 hours after the placement of catheters to allow the pigs to eat and drink water before feed and water were withheld during treatment procedures. At the end of the study (10 days), the skin sutures and the catheters were removed.

### 2.3 Treatments

#### 2.3.1 Control treatment

Feed and water were withheld from pigs for 12 hours during the treatment procedures. The feed and water withholding procedures for the pigs were the same for all 3 treatments. All wood shavings bedding was removed to prevent consumption by the pigs. When possible, feces were collected from the floor of pen and weighed during the treatment procedures. Respiratory rate, heart rate, and demeanor (agitation and restlessness) were monitored every 3 hours. Blood was collected at 0 (before treatment, T_0_) and 3 (T_3_), 6 (T_6_), 9 (T_9_), and 12 (T_12_) hours after the beginning of the treatment into a blood tube containing heparin as an anticoagulant (BD vacutainer, Beckton and Dickinson Company, Franklin Lakes, New Jersey). Blood was also collected in a tube containing no anticoagulant (BD vacutainer, Beckton and Dickinson Company, Franklin Lakes, New Jersey) at T_0_, T_6_, and T_12_ hours. Monitoring and sample collection procedures were the same among the 3 treatments.

#### 2.3.2 Fluid administration per rectum

The rectum was digitally evacuated to remove feces before fluid administration when necessary. A 10 French × 41 cm red rubber feeding tube (Feeding tube and urethral catheter, Covidien, Mansfield, Massachusetts) was lubricated and inserted into the rectum. The length of the feeding tube inserted into the rectum ranged from 25 to 30 cm. The catheter was secured and taped to the pig's tail.

Sterile polyionic crystalloid fluid (Plasma‐Lyte A 148 injection pH 7.4, Baxter Healthcare, Deerfield, Illinois) was administered continuously at 4.4 mL/kg/h for 12 hours using a fluid pump (Baxter Flo‐Gard Infusion pump, Baxter Healthcare, Deerfield, Illinois) and a coiled administration set (Large bore IV administration set with coiled administration set, Jorgensen Labs, Loveland, Colorado). The dose rate of 4.4 mL/kg/h (2 × maintenance rate) was based on previous studies and recommendations in ruminants and pigs.^9^, ^10^ Furthermore, the administration of polyionic fluids at dose rates ≥5 mL/kg/h can cause agitation and increased fecal water content in horses.^3^ Fluid administration was monitored every 15 minutes. Tolerance, as indicated by the level of discomfort to proctoclysis by pigs, was assessed subjectively by monitoring changes in behavior, including consistent agitation, restlessness, and straining or constant attempts to remove the red rubber tubing inserted in the rectum and physiologic variables (heart rate and respiratory rate) using a simple descriptive scoring system (Table 1). The scoring system was adapted and modified from previous reviews on the assessment of discomfort in humans undergoing proctoclysis^5^ and pain in pigs.^11^

**TABLE 1:** Descriptive discomfort scale assessing tolerance based on physiologic and behavioral changes for 5 pigs administered fluids by proctoclysis.

Score	Description
0	No overt signs of discomfort. Absence of avoidance to interaction with humans from a distance or closer in pen. The pig demonstrates expected lying behavior in lateral and sternal recumbency. Respiratory and heart rates are within reference ranges and similar before and after proctoclysis.
1	Moderate signs of discomfort. Periodic restlessness, agitation, or pacing. Periodic avoidance behavior during interactions with humans from a distance or in pen. Occasional hunched back and abdominal straining. Persistent moderate elevation above reference ranges of respiratory and heart rates during proctoclysis compared to before proctoclysis.
2	Severe signs of discomfort. Persistent restlessness, agitation, or pacing. Persistent avoidance and escape from interactions with humans from a distance or in pen. Persistent hunched back and abdominal straining. Attempts to bite and remove the red rubber tube. Dog sitting and bottom scooting. Constant high respiratory and heart rates during proctoclysis compared to before proctoclysis.

#### 2.3.3 Intravenous fluid administration

Intravenous administration of polyionic fluids was performed via 1 of the jugular venous catheters. The coiled administration set was secured on the back of the pigs with adhesive tape to prevent the pigs from chewing the set. Intravenous fluid type, administration rate, use of a fluid pump, and monitoring of fluid administration procedures were similar to fluid administration per rectum.

#### 2.3.4 Clinical laboratory measurements

The PCV and plasma total solids were measured using a microhematocrit reader (CritSpin microhematocrit centrifuge, Iris International, Inc, Westwood, Massachusetts) and an optic refractometer (Master refractometer, Atago USA, Inc, Bellevue, Washington) from samples collected in tubes with heparin as an anticoagulant collected at T_0_, T_3_, T_6_, T_9_, and T_12_ hours. Packed cell volume and plasma total solids were determined within 15 minutes after collection. Blood collected into tubes containing no anticoagulant at T_0_, T_6_, and T_12_ hours was centrifuged at 2800 × *g*, and serum was harvested. The serum was submitted to University of California Davis Veterinary Medical Teaching Hospital Clinical Pathology laboratory to determine serum total solids, sodium, chloride, ionized magnesium, potassium, blood urea nitrogen, creatinine, and albumin using a chemistry analyzer (Roche 501C, Roche Diagnostics, Indianapolis, Indiana) within 12 hours after collection. Serum total solids, albumin, creatinine, and blood urea nitrogen measurements were measured because these analytes might be altered with hemodilution or hemoconcentration. Sodium, chloride, ionized magnesium, and potassium concentrations were measured because these electrolytes are present in Plasma‐Lyte A 148.

### 2.4 Statistical analysis

The Shapiro‐Wilk test was used to test the data for normality. Mean ± SD was reported when data were normally distributed, whereas median (range) was reported when data were not normally distributed. Descriptive data, including body weight, dropout rate, and complications associated with the treatment procedures, were reported.

The effect of treatment (IV, RECT, and CONT) and time on clinical laboratory analyte measurements were compared using a 2‐factor repeated measures of analysis of variance (ANOVA). In the analysis of variance, treatment and time were considered fixed effects, whereas the subject (pig) was considered a random effect. When differences were present among treatments or time points, post hoc analysis was performed using the Tukey test. Because multiple analytes concentrations were determined from every sample collected, a 2‐factor multiple analysis of variance (MANOVA) was also performed to compare to the results of the ANOVA. In the MANOVA, treatment and time were also considered fixed effects, whereas the subject (pig) was considered a random effect. Interactions between treatment and time were considered for ANOVA and MANOVA analyses. Commercial statistical software was used to analyze the data (JMP Pro v16 software, SAS Institute, Cary, North Carolina). *P* < .05 was considered significant.

## 3 RESULTS

### 3.1 Complications and tolerance to proctoclysis

Data were normally distributed; therefore, mean ± SD was reported. All pigs were considered healthy before treatment based on clinical examination, CBC, and serum biochemical analysis. The mean ± SD weight for the pigs was 43.1 ± 5.7 kg. The mean duration from induction of anesthesia with tiletamine‐zolazepam to full recovery (pigs walking without assistance, eating, and drinking) from anesthesia was 4 hours. Assuming a half‐life of 4 hours for Plasma‐Lyte A,^7^ the predicted remaining Plasma‐Lyte A volume in the pigs' blood after a 3‐day washout period was less than 0.5 mL. Five out of 6 pigs completed the study. Therefore, results from 5 pigs are reported. One pig was euthanized after developing septicemia and endocarditis due to *Actinobacillus* spp., presumably secondary to catheter site infection, based on necropsy findings. Both intravenous catheters from the pig that was euthanized required a replacement after the first treatment (day 6 after the beginning of the procedures).

All pigs subjectively tolerated proctoclysis (median score 0), and no consistent agitation, restlessness, or straining to remove the red rubber tubing inserted in the rectum was observed during the procedures. There was minimal external leakage (<2 mL) of administered fluid per rectum. One (59.1 g), 2 (mean weight, 160.7 g), and 5 pigs (mean weight, 134.1 g) defecated during the RECT, CONT and IV treatment procedures, respectively. It was difficult to collect feces from pigs under proctoclysis when leakage was observed because of the liquid consistency. Due to the inconsistency of fecal output and difficulty in weighing feces during proctoclysis, further comparison of fecal output among treatments was not performed.

### 3.2 Effect of treatment and time on blood analytes

#### 3.2.1 Albumin, PCV, plasma total solids, and serum total solids

Treatment (*P* = .006) and interactions between treatment and time (*P* = .03) had a significant effect on albumin concentrations. Albumin concentrations at T_6_ were lower than T_0_ (least square mean of 3.9 vs 4.2 g/dL; mean difference = −0.28 g/dL; 95% CI = −0.52, −0.04) for the IV treatment. Albumin concentrations were not significantly different between any time points for the RECT treatment (*P* > .05); concentrations were 3.7, 3.7, and 3.7 g/dL at T_0_, T_6_, and T_12,_ respectively. Similarly, albumin concentrations were not significantly different (*P* > .05) at any time point for the CONT treatment; concentrations were 3.9, 4.0, and 4.1 g/dL at T_0_, T_6_, and T_12_, respectively. Time had no significant effect (*P* = .09) on albumin concentrations. The analysis of variance model (fixed effects) for albumin concentrations as a function of treatment, time, and interaction between treatment and time is summarized in Table 2.

**TABLE 2:** Analysis of variance table for fixed effects (treatment and time) on albumin concentrations in 5 pigs in a cross‐over design study.

Source	Number of variables	Degrees of freedom	*F*‐ratio	*P*‐value
Treatment	2	2	8.2	.006
Time	2	2	2.7	.09
Treatment × time	4	4	3.3	.03

*Note*: Treatment = Control, intravenous fluid administration, or per rectum administration. Time = 0 (before treatment), 6, and 12 hours after treatment began. Treatment × Time = first‐order interaction between treatment and time. Summary of fitness of model: adjusted *R* ^2^ for the model = 0.84. *P* < .05 significant. Random effects (subject = pig) *P*‐value was .06.

Treatment (*P* = .09), time (*P* = .99), and interactions between treatment and time (*P* = .42) had no significant effect on the PCV. Treatment (*P* = .09), time (*P* = .31), and interactions between treatment and time (*P* = .09) had no significant effect on plasma total solids. Time had a significant effect on serum total solids (*P* = .009), with total serum solids lower at T_6_ than at T_0_ (least square mean of 6.1 vs 6.3 g/dL; mean difference = −0.24 g/dL; 95% CI of mean difference = −0.42, −0.06). In contrast, time had no significant effect (*P* > .05) on serum total solids concentrations between T_0_ and T_12_ (least square mean of 6.3 vs 6.3 g/dL; mean difference = −0.07 g/dL; 95% CI = −0.11, −0.25) or T_6_ and T_12_ (least square mean of 6.1 vs 6.3 g/dL; mean difference = 0.17 g/dL; 95% CI = −0.01, −0.35). Treatment (*P* = .13) or interactions between treatment and time (*P* = .06) had no significant effect on serum total solids. Packed cell volume, plasma total solids, and serum total solids concentrations for the CONT, IV, and RECT treatments at different time points are summarized in Table 3.

**TABLE 3:** Least square mean (95% confidence interval) for PCV, plasma total solids, and serum total solids concentrations for the CONT, IV, and RECT treatments at different time points in 5 pigs enrolled in a cross‐over design.

Analyte	Treatment	T_0_	T_3_	T_6_	T_9_	T_12_
PCV (%)	CONT	31 (27, 35)	32 (28, 36)	32 (28, 36)	31 (27, 35)	32 (28, 37)
	IV	34.0 (30, 38)	28 (24, 32)	30 (26, 34)	32 (28, 36)	31 (27, 35)
	RECT	26 (22, 31)	31 (27, 35)	30 (26, 34)	27 (23, 32)	29 (26, 34)
Plasma total solids (g/dL)	CONT	6.0 (5.3, 6.7)	6.1 (5.4, 6.9)	6.3 (5.6, 7.0)	6.0 (5.2, 6.7)	6.4 (5.7, 7.1)
	IV	6.2 (5.5, 6.9)	4.5 (4.0, 5.4)	5.4 (4.7, 6.1)	5.6 (4.9, 6.3)	5.6 (4.9, 6.3)
	RECT	6.1 (5.4, 6.8)	6.3 (5.6, 7.0)	6.3 (5.6, 7.0)	6.3 (5.6, 7.0)	6.4 (5.7, 7.1)
Serum total solids (g/dL)	CONT	6.4 (6.0, 6.7)	–	6.2 (5.9, 6.6)	–	6.5 (6.2, 69)
	IV	6.2 (5.9, 6.6)	–	5.7 (5.3, 6.1)	–	5.9 (5.6, 6.3)
	RECT	6.4 (6.0, 6.7)	–	6.3 (6.0, 6.7)	–	6.3 (6.0, 6.7)

*Note*: All interaction comparisons between treatment and time were not significant (*P* > .05). CONT—control treatment. IV—IV treatment. RECT—per rectum infusion treatment. T_0_—time before treatment. T_3_, T_6_, T_9_, and T_12_, represent 3, 6, 9, and 12 hours after the beginning of the treatments at sample collection. Serum total solids were not determined at T_6_ and T_9_.

#### 3.2.2 Sodium, chloride, ionized magnesium, potassium, creatinine, and blood urea nitrogen

Treatment had a significant effect on sodium concentrations (*P* = .009). Sodium concentrations for RECT treatment were lower than the IV treatment (least square mean of 140 vs 145 mmol/L; mean difference = −4.9 mmol/L; 95% CI = −8.4, −1.5). Time (*P* = .57) or interactions between treatment and time (*P* = .40) had no significant effect on sodium concentrations. Treatment (*P* = .06), time (*P* = .79), and interactions between treatment and time (*P* = .97) had no significant effect on chloride concentrations. Treatment (*P* = .81), time (*P* = .48), and interactions between treatment and time (*P* = .53) had no significant effect on ionized magnesium concentrations. Treatment (*P* = .22), time (*P* = .51), and interactions between treatment and time (*P* = .31) had no significant effect on potassium concentrations.

Treatment (*P* = .46) and interaction between treatment and time (*P* = .09) had no significant effect on creatinine concentrations. In contrast, time (*P* = .0007) had a significant effect on creatinine concentrations. Creatinine concentrations at T_6_ were lower than at T_0_ (least square mean of 0.80 vs 0.87 mg/dL; mean difference = −0.07 mg/dL; 95% CI of mean difference = −0.1, −0.03). Treatment (*P* = .87) had no significant effect on blood urea nitrogen concentrations. In contrast, time (*P* = .03) and interactions between treatment and time (*P* = <.0001) had a significant effect on blood urea nitrogen concentrations. Blood urea nitrogen concentrations at T_12_ were lower than T_0_ (least square mean of 7.6 vs 8.8 mg/dL; mean difference = −1.2 mg/dL; 95% CI = −2.4, −0.02). Blood urea nitrogen concentrations at T_6_ (least square mean of 6.8 vs 11.2 mg/dL; mean difference = −4.4 mg/dL; 95% CI of mean difference = −7.2, −1.6) and T_12_ (least square mean of 5.0 vs 11.2 mg/dL; mean difference = −6.2 mg/dL; 95% CI of mean difference = −9.0, −3.4) were lower than at T_0_ for the IV treatment. The concentrations of sodium, chloride, ionized magnesium, potassium, creatinine, and blood urea nitrogen for the CONT, IV, and RECT treatments at different time points are summarized in Table 4.

**TABLE 4:** Least square mean (95% confidence interval) for sodium, chloride, ionized magnesium, potassium, creatinine, and blood urea nitrogen concentrations for the CONT, IV, and RECT treatments at different time points in 5 pigs enrolled in a cross‐over design.

Analyte	Treatment	T_0_	T_6_	T_12_
Sodium (mmol/L)	CONT	143 (141, 145)	142 (140, 144)	144 (142, 144)
	IV	146 (144, 148)	146 (143, 148)	145 (143, 148)
	RECT	141 (139, 143)	141 (139, 143)	140 (138, 142)
Chloride (mmol/L)	CONT	102 (99, 104)	102 (99, 104)	101 (99, 104)
	IV	104 (102, 107)	104 (102, 107)	104 (101, 106)
	RECT	100 (98, 103)	100 (99, 103)	100 (98, 103)
Ionized magnesium (mmol/L)	CONT	0.42 (0.39, 0.44)	0.43 (0.40, 0.45)	0.41 (0.39, 0.44)
	IV	0.42 (0.39, 0.44)	0.41 (0.39, 0.44)	0.42 (0.40, 0.45)
	RECT	0.41 (0.39, 0.44)	0.43 (0.41, 0.46)	0.43 (0.41, 045)
Potassium (mmol/L)	CONT	4.1 (3.9, 4.3)	4.1 (4.0, 4.4)	4.2 (3.9, 4.4)
	IV	4.1 (3.9, 4.3)	4.0 (3.7, 4.1)	4.0 (3.8, 4.2)
	RECT	4.1 (3.9, 4.4)	4.1 (3.9, 4.4)	4.4 (4.2, 4.6)
Albumin (g/dL)	CONT	3.9 (3.8, 4.1)	4.0 (3.8, 4.1)	4.1 (3.9, 4.2)
	IV	4.2 (4.0, 4.4)^a^	3.9 (3.7, 4.1)^b^	4.0 (3.9, 4.2)^a, b^
	RECT	3.7 (3.5, 3.8)	3.7 (3.5, 3.8)	3.7 (3.5, 3.8)
Creatinine (mg/dL)	CONT	0.9 (0.8, 1.0)	0.9 (0.7, 1.0)	0.9 (0.8, 1.0)
	IV	0.9 (0.8, 1.0)	0.8 (0.7, 0.9)	0.8 (0.7, 0.9)
	RECT	0.8 (0.7, 1.0)	0.8 (0.6, 0.9)	0.8 (0.7, 0.9)
Blood urea nitrogen (mg/dL)	CONT	7.2 (5.2, 9.2)	7.8 (5.8, 9.8)	9.4 (7.4, 11.4)
	IV	11.2 (9.2, 13.2)^c^	6.8 (4.8, 8.8)^d^	5.0 (3.0, 7.0)^d^
	RECT	8.0 (6.0, 10.0)	8.4 (6.4, 10.4)	8.4 (6.4, 10.4)

*Note*: Albumin and blood urea nitrogen concentrations for the IV treatment—comparisons (treatment and time interactions) with different superscripts are significantly different (*P* < .05) for the analyte. All other interaction comparisons between treatment and time were not significant (*P* > .05). CONT—control treatment. IV—IV treatment. RECT—per rectum infusion treatment. T_0_—time before treatment. T_6_ and T_12_ represent 6 and 12 hours after the beginning of the treatments at sample collection.

## 4 DISCUSSION

Continuous fluid infusion per rectum did not significantly affect serum biochemical analytes compared to intravenous administration of fluids in healthy pigs. Our results suggest that hemodilution due to the absorption of fluids from the rectal or distal colonic mucosa was not evident, inconsistent with our hypothesis. In contrast, serum albumin concentrations significantly decreased at T_6_ compared to T_0_ with the IV treatment, consistent with hemodilution. In horses, a reduction in albumin concentrations after 6 hours was reported only with intravenous administration of fluids but not with proctoclysis,^3^ consistent with our study. Changes in PCV, serum total solids, or plasma total solids concentrations among the treatments were not significant in our study. In contrast, PCV, and serum total solids decreased after per rectum administration of fluids in horses.^3^ The design differences between our study and the study in horses^3^ might explain the differences. In our study, the polyionic fluid administered per rectum and intravenous were similar, whereas a polyionic fluid was administered IV, but free water was administered per rectum in horses.^3^ The study in horses^3^ also reported that proctoclysis with isotonic fluids was not tolerated well, and absorption was variable compared to plain water. As expected, the changes in albumin concentrations in the CONT treatment were not significant at any time point.

The rectum in pigs is <5 cm in length, whereas the entire colon measures 5 m in length.^12^ Therefore, our study's red rubber feeding tube placement (25‐30 cm) was in the colon and rectum. The colonic enterocytes do not have villi, and colonic microvilli are less closely packed than the small intestine, but they play a significant role in the absorption of water, sodium, and other electrolytes.^13^, ^14^ The colonic pH in pigs is slightly acidic (6.8); therefore, we expected absorption of Plasma‐Lyte A 148 (pH, 7.4) to be higher due to increased solubility from ionization of the electrolytes. The colon and rectum in pigs have a volume capacity of 8.7 L.^12^ Using the average weight of the pigs in our study of 43.1 kg and a fluid rate of 4.4 mL/kg/h, the total volume delivered to the rectum and colon over 12 hours was 2.3 L. The total volume delivered and minimal external fluid leakage from the rectum observed during the monitoring of fluid administration suggest that the fluids were likely retained in the colon.

The anatomy of the colon in pigs is comparable to humans,^12^, ^15^, ^16^ and studies in humans demonstrated absorption of free water and electrolytes with proctoclysis.^4^, ^5^, ^6^ We administered a polyionic fluid instead of free water because our focus was to assess a crystalloid fluid type (Plasma‐Lyte A) routinely administered by proctoclysis in pigs managed in our clinical practice. We chose Plasma‐Lyte A because it is an isotonic, balanced crystalloid fluid type.^17^ The potential reasons for the lack of evidence of hemodilution in our study include the enrollment of healthy pigs for which feed and water were withheld during the treatment procedures. In contrast, the studies in humans that demonstrated an improvement in hydration or an increase in electrolyte concentrations were performed in sick, humans diagnosed with cancer. Variables such as PCV and serum total solids before treatment are likely higher in sick animals compared to healthy animals; therefore, the difference in concentrations of the analytes after administering fluids is likely detectable in sick animals. Withholding feed and water from the pigs in our study might have reduced fecal volume in the colon, allowing more retention of fluid administered per rectum.

A significant difference in the albumin concentrations for the IV treatment was detected between T_0_ and T_6_ but not between T_6_ and T_12_. A possible reason for this might be that the pigs enrolled in our study were healthy; therefore, normal physiological responses, such as increased fluid loss via urination, compensated for the hemodilution observed between T_0_ and T_6_. The significant effect of time on serum total solids, creatinine, and blood urea nitrogen or the higher sodium concentrations in the IV compared to the RECT treatments are of minimal practical clinical significance.

The pigs tolerated proctoclysis, and no complications associated with proctoclysis were recorded during the procedures. It is essential to note the intravenous catheter site infection associated with sepsis and endocarditis that we reported in 1 pig. The replacement of both catheters in this animal likely increased the likelihood of catheter site infection.

Although routinely performed in our clinical practice when venous access is unsuccessful, our study results suggest that proctoclysis does not demonstrate hemodilution comparable to the intravenous administration of polyionic fluids in healthy pigs. Despite being tolerated well in pigs, proctoclysis using polyionic fluids at a rate of 4.4 mL/kg/h might not be a sufficient alternative to the intravenous administration of fluids.

Our study limitations include a lack of performing a urinalysis. Urine collection was not performed because of the difficulty in maintaining urine collection bags in pigs for 12 hours; Despite removing all bedding, the pigs were observed chewing on the metal fencing on the pens while feed and water were withheld. Therefore, the pigs were likely to chew the urine collection bags. The scoring system adaptation described in our study was subjective, not validated, and the reliability of the scale is unknown. No further analysis of the scoring system analysis such as ordinal regression was performed because of the small sample size.^18^, ^19^ It is also important to note that the descriptive scale in our study focused on assessment of discomfort rather than pain during proctoclysis. Therefore, objective measurements for assessing pain in pigs including in cortisol and ACTH^11^ were not measured. We did not complete weighing the pigs before and after treatments because the pigs were housed close to horses. The squealing of the pigs during weighing startled the horses, which was unsafe because of attempts by horses to escape the stalls. Our study was performed in healthy pigs, and our results apply only to healthy pigs. Further studies should focus on proctoclysis in sick or dehydrated pigs, using different fluid solutions and analysis of serum biochemical analytes beyond 12 hours.

## 5 CONCLUSIONS

Pigs tolerated proctoclysis with a polyionic fluid, but the changes in analytes consistent with hemodilution were absent. Our results suggest that proctoclysis might not be an effective alternative to the intravenous administration of polyionic fluids in healthy, euvolemic pigs.

## CONFLICT OF INTEREST DECLARATION

Munashe Chigerwe serves as Associate Editor for the Journal Veterinary Internal Medicine. He was not involved in review of this manuscript. No other authors declare a conflict of interest.

## INSTITUTIONAL ANIMAL CARE AND USE COMMITTEE (IACUC)

Approved by the University of California, Davis IACUC, protocol: #21935.

## HUMAN ETHICS APPROVAL

The authors declare that human ethics approval was not needed for this study.
